# The post-translational modification of the *Clostridium difficile* flagellin affects motility, cell surface properties and virulence

**DOI:** 10.1111/mmi.12755

**Published:** 2014-09-15

**Authors:** Alexandra Faulds-Pain, Susan M Twine, Evgeny Vinogradov, Philippa C R Strong, Anne Dell, Anthony M Buckley, Gillian R Douce, Esmeralda Valiente, Susan M Logan, Brendan W Wren

**Affiliations:** 1 London School of Hygiene and Tropical MedicineKeppel Street, London, WC1E 7HT, UK; 2 Vaccine Program, Human Health Therapeutics Portfolio, National Research CouncilOttawa, ON, K1A 0R6, Canada; 3 Department of Life Sciences, Imperial CollegeLondon, SW7 2AZ, UK; 4 Institute of Infection, Immunity and Inflammation, Glasgow Biomedical Research Centre, University of GlasgowGlasgow, UK

## Abstract

*Clostridium difficile* is a prominent nosocomial pathogen, proliferating and causing enteric disease in individuals with a compromised gut microflora. We characterized the post-translational modification of flagellin in *C. difficile* 630. The structure of the modification was solved by nuclear magnetic resonance and shown to contain an *N*-acetylglucosamine substituted with a phosphorylated *N*-methyl-l-threonine. A reverse genetics approach investigated the function of the putative four-gene modification locus. All mutants were found to have truncated glycan structures by LC-MS/MS, taking into account bioinformatic analysis, we propose that the open reading frame CD0241 encodes a kinase involved in the transfer of the phosphate to the threonine, the CD0242 protein catalyses the addition of the phosphothreonine to the *N*-acetylglucosamine moiety and CD0243 transfers the methyl group to the threonine. Some mutations affected motility and caused cells to aggregate to each other and abiotic surfaces. Altering the structure of the flagellin modification impacted on colonization and disease recurrence in a murine model of infection, showing that alterations in the surface architecture of *C. difficile* vegetative cells can play a significant role in disease. We show that motility is not a requirement for colonization, but that colonization was compromised when the glycan structure was incomplete.

## Introduction

The intestinal pathogen *Clostridium difficile* is a strictly anaerobic, Gram-positive bacterium. In recent years it has become a huge burden both economically and to human health due to its global spread throughout health care environments, where it causes disease ranging from mild diarrhoea to life threatening pseudomembranous colitis (Loo *et al*., 2005; Goorhuis *et al*., 2007; Kuijper *et al*., 2007). Its ability to differentiate into endospores, a metabolically dormant and highly robust cell form, is essential in its transmission as this allows it to survive external stresses such as desiccation, osmotic shock, and contact with chemicals such as disinfectants (Lawley *et al*., 2010; Deakin *et al*., 2012). Two large clostridial toxins, TcdA and TcdB are considered the main virulence factors associated with disease symptoms, their production leading to the disruption and eventual destruction of the intestinal epithelium (Voth and Ballard, 2005). The generation of at least one of these two potent toxins is essential for the pathogenesis of this organism (Lyras *et al*., 2009; Kuehne *et al*., 2010); however, other properties of *C. difficile* contribute to its success as a pathogen by promoting the colonization of the host, which may be influential in disease recurrence.

Bacterial surface-associated proteins are often involved in host colonization and immune evasion (Pizarro-Cerda and Cossart, 2006). *C. difficile* produces many such proteins, including peritrichous flagella, a paracrystalline S-layer, cell wall proteins and a putative sortase and sorted proteins (Hennequin *et al*., 2003; Eidhin *et al*., 2006; Kirby *et al*., 2009; Reynolds *et al*., 2011). Flagella are the organelles of bacterial propulsion and have been found to be important for the penetration of the mucous layer and adherence to the gut mucosa in enteric pathogens (Liu *et al*., 1988; Grant *et al*., 1993), although in *C. difficile* the flagella are not essential for virulence and colonization of the hamster or murine model (Dingle *et al*., 2011; Aubry *et al*., 2012; Baban *et al*., 2013).

Flagellin is the main protein component of the flagella; it forms the long whip-like filament which when rotated by the basal subunits of the structure causes motility. Some bacteria produce ‘simple’ flagella filaments which are composed of a single flagellin, while others produce ‘complex’ flagella filaments made up of multiple flagellins (Logan, 2006). In a number of Gram-positive and Gram-negative bacteria the flagellin is post-translationally modified with an O-linked oligosaccharide at serine and/or threonine residues on the highly variable, surface-exposed domains of the protein (Logan, 2006). Enzymes, often encoded alongside the flagella genes, transfer these post-translational modifications (PTMs) specifically to the flagellin. The nature of this specificity is yet to be determined as no consensus sequence is recognized by these enzymes. In many bacteria these flagellin PTMs are essential for motility and mutants of the PTM enzymes are not only unable to modify their flagellin but are also unable to secrete the flagellin from the cell, as has been described in *Campylobacter jejuni*, *Helicobacter pylori*, *Caulobacter crescentus*, *Aeromonas caviae* and *Shewanella oneideinsis* (Ewing *et al*., 2009; Asakura *et al*., 2010; Faulds-Pain *et al*., 2011; Parker *et al*., 2012; Sun *et al*., 2013). However, this is not the case for all bacteria, for example in *Pseudomonas aeruginosa* motility is unaffected when flagellin modification genes are mutated (Schirm *et al*., 2004; Verma *et al*., 2006).

*C. difficile* produces a simple flagella filament with a single flagellin which is post-translationally modified. As with most bacterial O-linked PTM systems, the genes encoding the modification enzymes are colocalized with those encoding the target protein. In *C. difficile* strains these enzymes are thought to be encoded among the flagella structural and regulatory genes, between the flagellin gene, *fliC* and the putative flagella basal body rod gene, *flgB* (Stabler *et al*., 2009).

**Fig 1:**
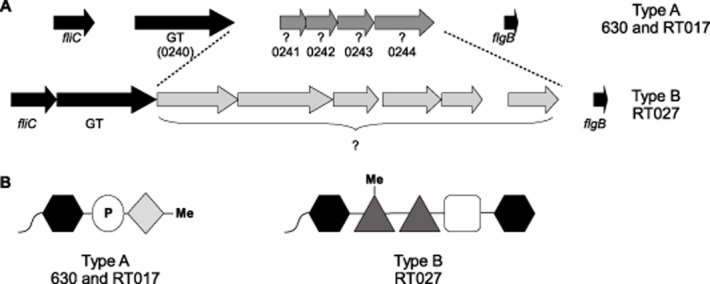
Schematic diagram illustrates the alternative flagellin modification gene loci type A and type B.A. The flagella structural genes *fliC* and *flgB* flank the genes predicted to be involved in flagellin modification in the ribotype 012 strain 630 and the ribotype 027 strains. The gene adjacent to *fliC* in both loci is a predicted glycosyltransferase (GT) and the ORFs in grey are the remaining putative modification genes, predicted to be involved in flagellin modification.B. The predicted modification structures of the type A 630/RT017 strains and the type B RT027 strains. The black hexagon represents a HexNAc, the white circle represents a phosphate group, Me denotes a methyl group, dark gray triangles represent a deoxyhexose and the white square a heptose, the light gray diamond represents an unidentified structure.

It has been found that the *C. difficile* flagellin can be modified with at least two oligosaccharide structures, herein referred to as type A and type B, and that these structural variations are strain-specific and reflected in variations at the genome level (Fig. 1; Twine *et al*., 2009). The coding sequence (CDS) immediately downstream of *fliC* is conserved in the type A and type B strains and encodes a putative glycosyltransferase (GT) (Fig. 1A), which is essential for the modification of the flagellin and motility but not flagellin secretion or filament formation (Twine *et al*., 2009). The sugar associated with the flagellin protein in both type A and type B oligosaccharide modifications, has been identified by mass spectrometry to be an *N*-acetyl hexosamine (HexNAc) (Twine *et al*., 2009), suggesting that this first sugar is transferred to the flagellin by this common GT. Beyond this initial sugar neither the modification genes nor the structure of the PTMs are conserved in the type A and type B strains (Smith *et al*., 1981; Verma *et al*., 2006). The type A flagellin modification was previously proposed to be a methylated aspartic acid attached to the initial HexNAc via a phosphate bond in the non-epidemic strain 630 (Twine *et al*., 2009). The type B structure was identified among BI-I/NAP-I/ ribotype 027 epidemic strains. The oligosaccharide structure of these modifications is complex and consists of multiple monosaccharide residues which includes a HexNAc moiety through which the glycan is linked to the protein as well as two deoxyhexose residues and a heptose (Fig. 1B; Twine *et al*., 2009; Hitchen *et al*., 2010).

It has been hypothesized that as the type B PTM was identified in BI-I/NAP-I/ ribotype 027 outbreak strains, while the type A PTM was found in a non-epidemic strain, the type B PTMs could be contributing to the hypervirulence of these organisms (Stabler *et al*., 2009; Twine *et al*., 2009). In this study however we identified the presence of the type A loci in the toxin A negative epidemic ribotype 017 (RT017) strains of *C. difficile*, indicating that the relative importance of the type A and type B modifications might not be easily defined. We investigate the flagellin type A PTMs in both the 630 strain and an epidemic RT017 strain M68. A definitive structure for this modification was determined by NMR spectroscopy and the role of the putative modification genes were assessed. We also investigated the role of motility and flagellin modification in the colonization of C57BL/6 mice, finding that motility is not a requirement but that colonization was compromised when the glycan structure was incomplete.

## Results

### Orthologues of the type A modification gene cluster are found only in *P. aeruginosa* PA01 strains

Molecular typing and sequence analysis has revealed that there are at least five clonal lineages of *C. difficile*, designated clades one to five (He *et al*., 2010; Stabler *et al*., 2012), and that all have been involved in outbreaks of *C. difficile*-associated disease. Utilizing ACT comparisons; putative flagellin modification gene loci were compared among the five clonal lineages of *C. difficile* strains. Orthologues of the 630 type A modification genes were found in only one of these lineages which includes the epidemic RT017 strains.

To determine whether the type A modification genes are commonly found associated with flagella genes, BLASTP analysis was carried out with the sequences of strain 630 open reading frames (ORFs) CD0241, CD0242, CD0243 and CD0244. While orthologues of the ORFs CD0241 and CD0242 were occasionally identified together in other genomes (such as *Helicobacter hepaticus*) they were not associated with any known structural orthologues such as the flagella. In fact it was only in strain PA01 of *P. aeruginosa* where orthologues of CD0241, CD0242 and CD0243 were clustered together and like *C. difficile* they were found within the flagella genetic locus. It was noted previously that the flagellin modification of the PA01 flagellin was similar to that of *C. difficile* 630 (Twine *et al*., 2009), containing a methylated amino acid (in this case a tryptophan) attached via a phosphate bond to a deoxyhexose sugar on the surface of the flagellin (Verma *et al*., 2006). The genes PA1088, PA1089 and PA1090, located directly upstream of the flagellin GT and flagellin genes in *P. aeruginosa* PA01 strains, are orthologues of CD0243, CD0241 and CD0242 respectively and are involved in the modification of the PA01 flagellin. Their amino acid similarity, location and the similar structure of the modification indicate that these genes may have a similar function in *C. difficile*. No orthologue of the CD0244 ORF was found to be co-located with the other three ORFs in *P. aeruginosa* or any other organism and BLASTP searches only identified hypothetical proteins.

### NMR analysis of the type A flagellin solves the structure of its post-translational modification

**Fig 2:**
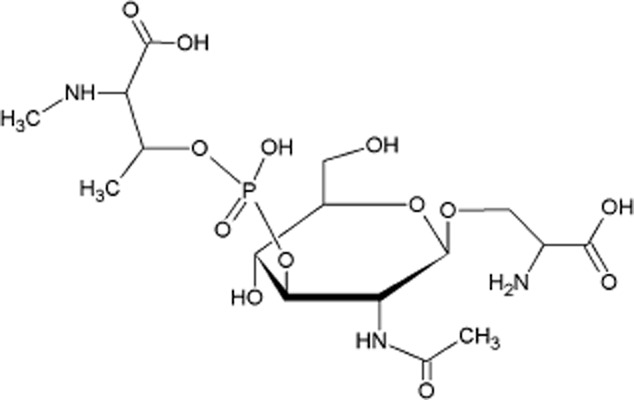
Schematic diagram illustrates the structure of the *C. difficile* 630 flagellar glycan shown in O-linkage to a serine residue, solved by NMR.

The structure of the *C. difficile* 630 flagellin glycan has been previously studied by LC-MS/MS and defined as a HexNAc sugar carrying a phosphate linked to a moiety which was suggested to be a methylated aspartic acid (Fig. 1B). This glycan was attached to the flagellin via the HexNAc at five sites (S^141^, S^174^, T^183^, S^188^ and S^205^ [S = serine and T = threonine]) (Twine *et al*., 2009). However, it has since been noted that at a mass of 115 Da the methylated amino acid is too small to be a methyl-aspartic acid (129 Da). In order to solve the structure of this modification, nuclear magnetic resonance (NMR) analysis of the flagellin glycan was carried out. Glycopeptides were released from the purified glycoprotein by Proteinase K treatment and isolated using size-exclusion and anion-exchange chromatography. Enzymatic hydrolysis did not cleave all amino acids and the product represented a mixture of glycosylated short peptides. NMR analysis using 2D spectra (gCOSY, TOCSY, ROESY, ^1^H-^13^C HSQC, ^1^H-^13^C HMBC, ^1^H-^31^P HMQC) indicated that the single HexNAc monosaccharide is a N-acetyl-β-glucosamine (GlcNAc) (all vicinal coupling constants 8–10 Hz), linked to a serine residue on the flagellin protein (Fig. 2). GlcNAc was phosphorylated at O-3, which caused a downfield shift of H/C-3 signals (Table 1). Additional signals were present, belonging to HOOC-CHX-CHX-CH_3_ fragment and a methyl group (Table 1). HMBC correlations between H/C of the methyl group and H/C-2 of this fragment indicated that methyl group was linked to the C-2 through a heteroatom. By the signal position of the C-2 at 70.5 ppm this heteroatom was most probably nitrogen. A downfield shift of the C-2 signal from its expected position of approximately 55 ppm was thus caused by N-methylation. C-3 carried a hydroxyl group which followed from the position of the C-3 signal at 73 ppm. The H-3 signal of the amino acid was shifted to 4.5 ppm due to phosphorylation. Overall this represents phosphorylated *N*-methylthreonine, in agreement with MS data indicating the mass of the glycosyl moiety at 398.1 amu (Fig. 2). Absolute l-configuration and identity of the *N*-methylthreonine was confirmed by GC of its acetylated ester with optically pure 2-octanol.

**Table 1:** NMR data (δ, ppm; Varian INOVA 500 MHz, 25°C) for the *C. difficile* 630 glycopeptide

	H/C 1	H/C 2	H/C 3	H/C 4	H/C 5	H/C 6	Me
GlcNAc	4.65	3.85	4.17	3.63	3.51	3.79; 3.93	
	100.1	55.6	79.9	70.5	76.6	61.6	
*N*-Me-l-Thr		3.52	4.51	1.41			2.76
	172.0	70.5	73.0	19.6			33.9

Exact mass of glycosyl cation 398.109035.

NAc signals C-1 176.1; H/C-2 2.08/23.8 ppm. ^31^P at −0.5 ppm.

The linkage between *N*-methylthreonine and GlcNAc was determined from the ^1^H-^31^P HMQC spectrum which showed correlations between H-3 of GlcNAc and H-3 of the *N*-methylthreonine and the same ^31^P signal at −0.5 ppm. To summarize, NMR revealed that the 630 glycan is formed of a single GlcNAc linked to a phosphorylated *N*-methyl-l-threonine at the oxygen at C3 of the sugar (Fig. 2).

### The type A modification genes are essential for motility in strain 630

**Fig 3:**
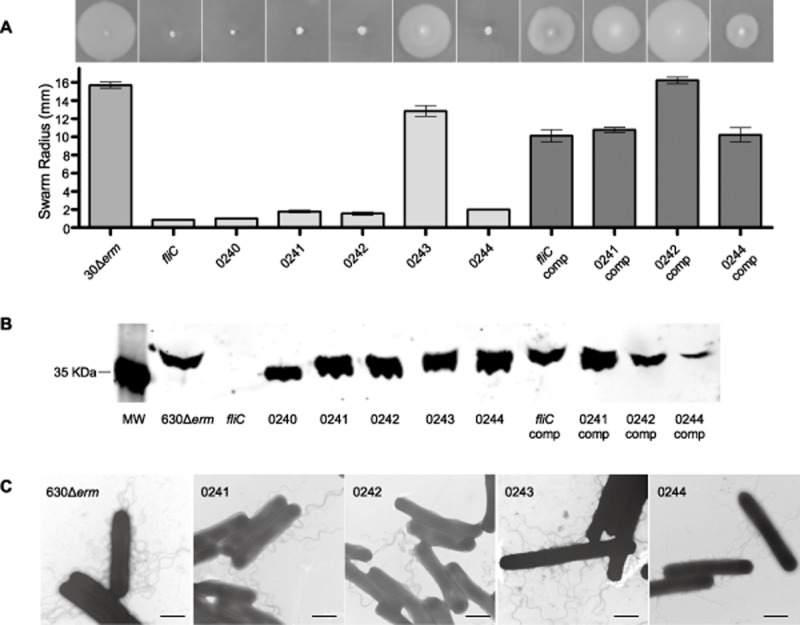
Motility and flagella production in 630 putative modification mutants.A. Motility assays of the 630Δ*erm* parental strain compared to the *fliC* Clostron mutant and the mutations in the predicted modification genes were carried out in CDMM containing 0.3% Difco-bacto agar. The *fliC* mutant and the initial GT mutant were non-motile, as described previously, as were mutations in the ORFs CD0241, CD0242 and CD0244. These were all restored to motility by complementation.B. Flagellin from 630Δ*erm* and the modification mutants were probed with an anti-FliC antibody by Western blot (MW = molecular weight, gene names and numbers below indicate mutation, comp = *in trans* complementation of mutant). All strains with the exception of the *fliC* mutant were found to produce flagellin, although the mass of the protein varied.C. The parental strain 630Δ*erm* and the mutants of CD0241, CD0242, CD0243 and CD0244 were observed by TEM, the black scale bar in each image represents a length of 1 μm. All of the mutants appeared to have flagella associated with the surface of the cells.

Flagellin modification is essential to motility in many organisms which modify their flagellins including *C. difficile*, in which mutation of the GT believed to transfer the first sugar to the flagellin protein causes a loss of motility in 630Δ*erm* (Twine *et al*., 2009). To determine whether the other putative type A modification genes are required for *C. difficile* motility, mutations in the 630 ORFs CD0241, CD0242, CD0243 and CD0244 were generated by Clostron mutagenesis in 630Δ*erm* (Table 2 for all strains and plasmids). These mutants were assessed for motility by different methods as have been previously described (Twine *et al*., 2009; Baban *et al*., 2013), the most reproducible and quantifiable results were achieved on *C. difficile* minimal media (CDMM) motility plates. In all assays the ORFs CD0241, CD0242 and CD0244 were found to be essential for the motility of *C. difficile* 630Δ*erm*, with the normal swimming phenotype of the parental strain being completely abolished in these mutants, this was also observed in mutations of *fliC* and the initial GT CD0240 as was previously described (Fig. 3A; Twine *et al*., 2009). The non-motile phenotypes were all restored on complementation *in trans* (Fig. 3A), although the phenotypes of the *fliC*, CD0241 and CD0244 mutants were not restored to wild type levels. The mutant of CD0243 formed smaller swarms than the parental strain; however this difference was not statistically significant (Fig. 3A).

**Table 2:** Bacterial strains and plasmids

Strain/plasmid	Genotype or description	Reference(s) or source
**Strains**		
*E. coli*		
Top10	F^−^ *mcrA* Δ(*mrr-hsdRMS-mcrBC*) φ80*lacZ*ΔM15 Δ*lacX74 nupG recA1 araD139* Δ(*ara-leu*)*7697 galE15 galK16 rpsL*(Str^r^) *endA1* λ^−^	Invitrogen
CA434	*E. coli* HB101 [F^−^ *mcrB mrr hsdS20*(r_B_^−^ m_B_^−^) *recA13 leuB6 ara-14 proA2 lacY1 galK2 xyl-5 mtl-1 rpsL20*(Sm^r^) *glnV44* λ^−^] containing plasmid R702	Williams *et al*. (1990)
*C. difficile*		
630Δ*erm*	Derivative of 630 strain, erythromycin sensitive	Hussain *et al*. (2005)
630Δ*erm-fliC*	*fliC*::CT insertion mutant derived from 630Δ*erm*	Twine *et al*. (2009)
630Δ*erm*-0240	0240 mutant derived from 630Δ*erm* by clostron insertion	Twine *et al*. (2009)
M68Δ*erm*	M68Δ*erm*: Derived from M68 by deletion of *ermB*	Faulds-Pain and Wren (2013)
M68Δ*fliC*	M68Δ*fliC*: Derived from M68 by deletion of *fliC*	Faulds-Pain and Wren (2013)
630Δ*erm-*0241::CT	0241 mutant derived from 630Δ*erm* by clostron insertion	This study
630Δ*erm*-0242::CT	0242 mutant derived from 630Δ*erm* by clostron insertion	This study
630Δ*erm*-0243::CT	0243 mutant derived from 630Δ*erm* by clostron insertion	This study
630Δ*erm*-0244::CT	0244 mutant derived from 630Δ*erm* by clostron insertion	This study
M68Δ*erm*-0242::CT	0242 mutant derived from M68Δ*erm* by clostron insertion	This study
M68Δ*erm*-0243::CT	0243 mutant derived from M68Δ*erm* by clostron insertion	This study
630Δ*erm*-0241 Δ*fliC*	Double mutant *fliC* deletion mutant derived from 630Δ*erm-*0241::CT	This study
630Δ*erm*-0242 Δ*fliC*	Double mutant *fliC* deletion mutant derived from 630Δ*erm*-0242::CT	This study
630Δ*erm*-0244 Δ*fliC*	Double mutant *fliC* deletion mutant derived from 630Δ*erm*-0244::CT	This study
**Plasmids**		
pMTL84151	*E. coli* – *C. difficile* shuttle plasmid (pCD6;*catP*; ColE1+*tra*)	Heap *et al*. (2009)
p*fliC*_comp	pMTL84151, with *C. difficile* 630 *fliC* and its native promoter	This study
pMTL84153	*E. coli* – *C. difficile* shuttle plasmid (pCD6;*catP*; ColE1+*tra*; *fdx* promoter)	Heap *et al*. (2009)
p0241_comp	pMTL84153 with CD0241 cloned behind the *fdx* promoter	This study
p0242_comp	pMTL84153 with CD0242 cloned behind the *fdx* promoter	This study
p0244_comp	pMTL84153 with CD0244 cloned behind the *fdx* promoter	This study
pMTL007C-E2-CD0241	Clostron plasmid retargeted to CD0241 at 231/232s (DNA 2.0)	This study
pMTL007C-E2-CD0242	Clostron plasmid retargeted to CD0242 at 156/157s (DNA 2.0)	This study
pMTL007C-E2-CD0243	Clostron plasmid retargeted to CD0243 at 309/310a (DNA 2.0)	This study
pMTL007C-E2-CD0244	Clostron plasmid retargeted to CD0244 at 556/557a (DNA 2.0)	This study
pMTLAFP*fliC*	pMTL82151 with + /− 630*fliC* 1200 bp	Faulds-Pain and Wren (2013)

It was previously observed by Twine and co-workers that despite being non-motile, the GT CD0240 mutant still produced polymerized flagella filaments, although they were truncated and fewer in number compared to the parental strain (Twine *et al*., 2009). To determine whether this was also true of the CD0241, CD0242, CD0243 and CD0244 putative flagellin modification mutants, we extracted the cell surface-associated proteins by glycine extraction and probed them with an anti-FliC antibody on a Western blot. All mutants were found to still produce flagellin, although its size varied (Fig. 3B). This is likely to be caused by alterations in the modifications leading to changes in the size of the protein.

Individual cells were visualized by negative stain transmission electron microscopy (TEM) by which the presence of flagella filaments associated with the surface of the cells in 630Δ*erm* and the CD0241, CD0242, CD0243 and CD0244 (Fig. 3C). On further analysis of these electron micrographs it was observed that the flagella of the non-motile mutants of CD0241, CD0242 and CD0244 appeared to clump together, which was not the case for the motile parental strain, 630Δ*erm*, or the motile mutant CD0243 whose flagella filaments were distributed evenly around the cell.

### Mutations in the type A modification genes cause alterations of the PTM

**Fig 4:**
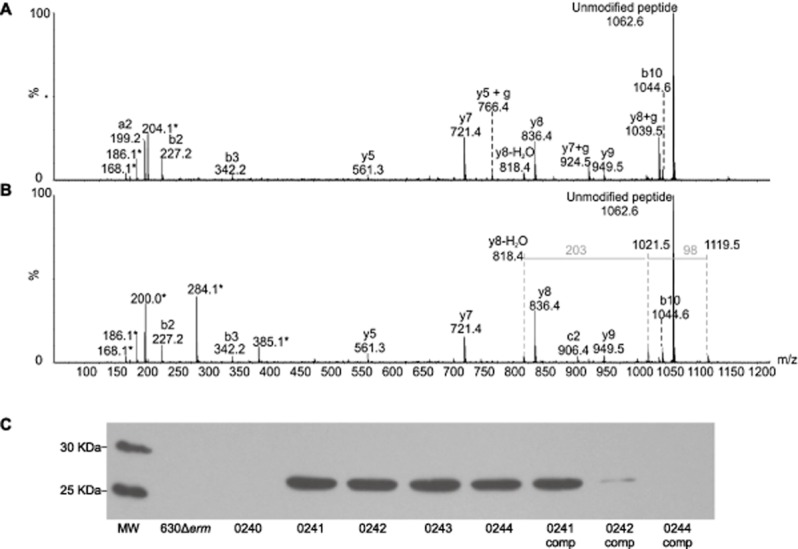
Mass spectrometry analyses of *C.* *difficile* flagellin tryptic glycopeptides from strain 630Δ*erm* CD0243 mutant. Peptide MS/MS spectra were obtained from in gel digestion of protein bands from glycine extraction of whole cells.A. MS/MS spectrum of *C. difficile* flagellin glycopeptide LLDGTSSTIR. The unmodified peptide ion was observed at m/z 1062, with low intensity peptide type y and b ions confirming the peptide sequence. A moderately intense HexNAc oxonium ion was observed at m/z 204, with sequential dehydration giving rise to glycan related ions at m/z 186 and 168. Glycan related fragment ions are indicated with an asterisk.B. MS/MS spectrum of *C. difficile* flagellin glycopeptide LLDGTSSTIR, modified with a glycan of 384 Da. The MS/MS spectrum shows the typical peptide fragment ions and an additional, with a glycan oxonium ion at m/z 385. Glycan related ions were also observed at m/z 284, 186 and 168. In addition, in the higher m/z region of the spectrum neutral losses of 98 and 203 Da were observed. Together these data suggest the 384 Da glycan moiety is similar to the 398 Da wild type sugar, with the absence of a methyl group. C, Flagellin from 630Δ*erm* and the modification mutants were analysed by Western blot, probing with an anti-β-*O*-GlcNAc antibody (MW = molecular weight, gene numbers below indicate mutation, comp = *in trans* complementation of mutant). With the exception of the CD0240 mutant, the flagellin of all mutants was found react with the anti-β-*O*-GlcNAc antibody, however the parental strain 630Δ*erm* and the CD0244 complement did not.

Disruption of the putative GT gene, CD0240, was previously reported to result in an inability to attach glycan to the sites of modification on the flagellin (Twine *et al*., 2009). In order to characterize the role of the remaining type A modification genes, the flagellin modifications of the CD0241, CD0242, CD0243 and CD0244 mutants were examined by Mass Spectrometry. Flagellin was isolated from wild type and mutant strains by shearing or glycine extraction and proteins were separated by SDS-PAGE. Gel bands corresponding to flagellin were excised, digested with trypsin and analysed by nLC-MS/MS. The corresponding MS/MS spectra were sequenced *de novo*, and the identified glycopeptides are listed in Table 3. Tandem mass spectrometry analyses of flagellin tryptic digests from the CD0241 mutant showed glycopeptides to be modified with a single HexNAc moiety. No peptides from the CD0241 mutant harboured the wild-type 398 Da glycan. Flagellin isolated from the CD0242 mutant also only harboured glycopeptides modified with a single HexNAc moiety. In contrast, flagellin isolated from the CD0243 mutant showed variable glycan modifications. A number of glycopeptides were modified with a single HexNAc moiety (Fig. 4A), and a number of peptides modified with a 384 Da glycan (Fig. 4B). The MS/MS spectrum of this structure showed typical peptide fragmentation and a glycan oxonium ion at m/z 385. Glycan-related fragment ions were also observed at m/z 284, 186 and 168, identical to the fragmentation pattern of the wild-type sugar (Twine *et al*., 2009). In the high m/z region of the MS/MS spectrum, neutral losses of 98 and 203 were observed, suggesting the 384 Da sugar is closely related to the wild type 398 Da sugar. The sugar structures appear to differ by 14 mass units, which indicates absence of the methyl group in the sugar of the CD0243 mutant strain. *De novo* sequencing of MS/MS spectra of flagellin from the CD0244 mutant showed a mixture of glycopeptides, modified with either a single HexNAc moiety, or the 398 Da wild-type sugar.

**Table 3:** Summary of glycan modifications of *C. difficile* 630 flagellin glycosylation mutants

Strain/plasmid	Detected glycopeptides (precursor m/z)	Glycan masses and predicted composition (Da)
630Δ*erm*	Various peptides	398
CD0241 mutant	LLDGTSSTIR (633.3^2+^)	203
	TMVSSLDAALK (669.8^2+^)	203
CD0241 mutant p0241_comp	AGGTTGTDAAK (577.3^2+^)	203
	TMVSLDAALK (697.4^2+^)	203
	TMVSLDAALK (775.3^2+^)	398 (203-80-115)
	LLDGTSSTIR (633.3^2+^)	203
	LLDGTSSTIR (730.8^2+^)	398(203-80-115)
CD0242 mutant	TMVSSLDAALK+ox (677.8^2+^)	203
CD0242 mutant p0242_comp	TMVSSLDAALK (679.3^2+^)	203
		384(203-80-101)
		398(203-80-115)
CD0243 mutant	LLDGTSSTIR (633.3^2+^)	203
	LLDGTSSTIR (732.8^2+^)	384(203-80-101)
	TMVSSLDAALK (679.3^2+^)	203
	TMVSSLDAALK (724.2^2+^)	384(203-80-101)
CD0244 mutant	TMVSSLDAALK (679.3^2+^)	203
	TMVSSLDAALK (775.5^2+^)	398(203-80-115)
	LLDGTSSTIR (730.8^2+^)	398(203-80-115)
CD0244 mutant p0244_comp	LLDGTSSTIR (730.8^2+^)	398(203-80-115)
	TMVSSLDAALK (775.5^2+^)	398(203-80-115)

*In trans* complementation of CD0241, CD0242 or CD0244 mutants either partially or fully restored modification of flagellin with the 398 Da wild type sugar (Table 3). For example, flagellin isolated from the complemented CD0241 mutant, showed glycopeptides modified with either HexNAc or the wild-type sugar. Flagellin isolated from the CD0242 complement harboured peptides modified with a 203 Da sugar, 384 Da sugar or the 398 Da wild-type sugar. Complementation of the CD0244 mutant appeared to fully restore wild type glycosylation.

Surface layer extracts of 630Δ*erm* and the modification mutants were analysed by Western blot with an anti-β-*O*-GlcNAc antibody and confirmed the NMR structure that the initial sugar is a GlcNAc (Fig. 4C). Interestingly, there was no interaction of this antibody with the 630Δ*erm* flagellin but it did bind to the flagellins of the CD0241, CD0242, CD0243 and CD0244 mutants, suggesting that the further modifications block the epitope for antibody binding to the GlcNAc residue in the parental strain glycan. As predicted no binding of the antibody to the flagellin of the CD0240 mutant, which cannot transfer glycan to the flagellin, was observed.

### Alterations in the flagella PTM causes cell aggregation

**Fig 5:**
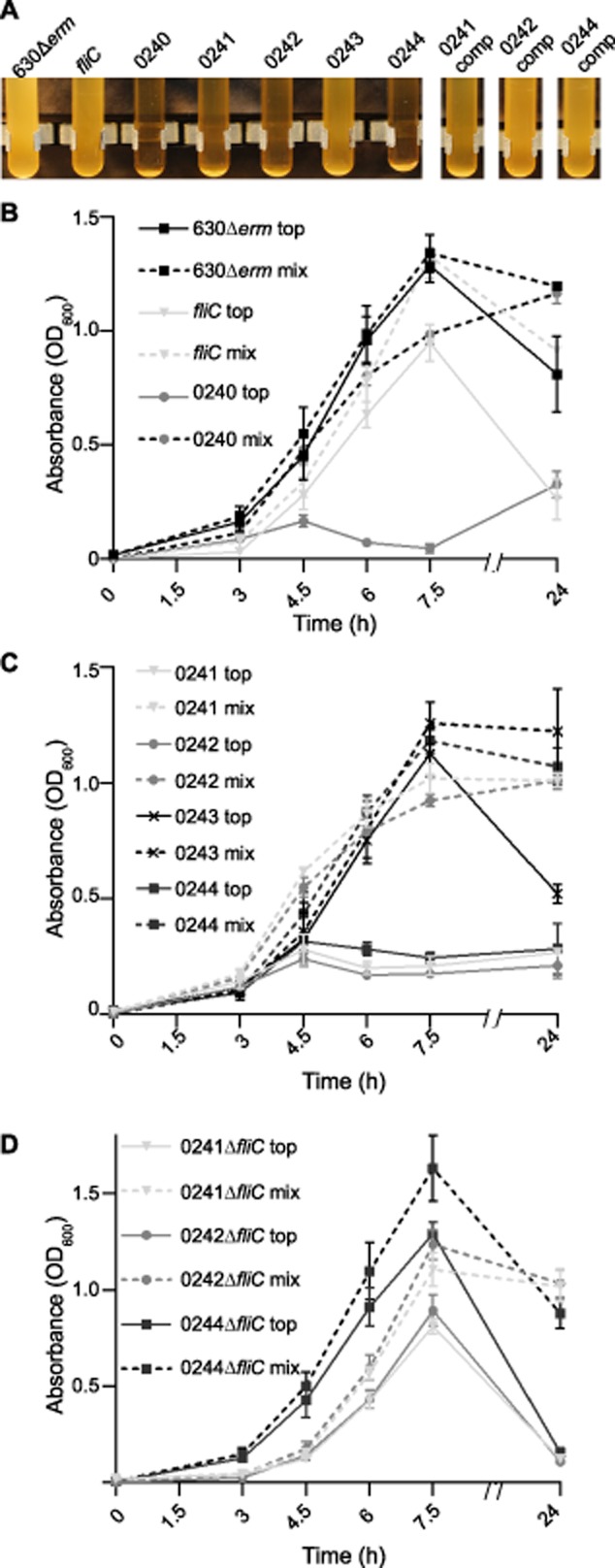
Sedimentation of the modification mutants.A. Mutations in the modification genes led cells to sediment in liquid culture with the exception of CD0243, this phenotype was restored by complementation *in trans*.B and C. This was quantified during growth by measuring the OD_600_ of the top 1 ml of culture (top – continuous lines) and a well mixed culture (mix – broken lines), demonstrating sedimentation in the CD0240, CD0241, CD0242 and CD0244 mutants, confirming the observations in A.D. The sedimentation in double mutants of CD0241 and *fliC*, CD0242 and *fliC* and CD0244 and *fliC* were also quantified. These double mutants had a growth profile similar to the *fliC* clostron mutant (B).

When grown in liquid culture we observed that the CD0240, CD0241, CD0242 and CD0244 mutants all settle out of the suspension and formed sediment at the bottom of the growth vessel, while cultures of the parental strain 630Δ*erm* and the CD0243 mutant remained in suspension (Fig. 5A). This phenotype was restored upon complementation *in trans* (Fig. 5A). As all mutants that formed sediment were also non-motile it was hypothesized that these two phenotypes could be related and that loss of motility led to sedimentation. However, when the non-motile aflagellate *fliC* mutant was grown in liquid it behaved as the motile parental strain and remained in suspension, suggesting that loss of motility alone cannot account for sedimentation of the modification mutants (Fig. 5A).

In order to quantify this phenotype an assay was developed which allowed us to determine whether the cells settled out during growth or at stationary phase and whether the growth rate was affected by this phenotype. For this we measured both the optical density (OD) of a well mixed culture (referred to here as a mixed culture) in order to assess the overall growth rate of the strains and the OD_600_ from the top 1 ml of an undisturbed culture (referred to here as an unmixed culture), which when compared to the OD_600_ of the mixed culture would indicate whether the cells were coming out of suspension. As the process of measuring these OD_600_s involves mixing and altering the overall volume of the cultures, it meant that two separate cultures were required for the mixed and unmixed measurements and that new cultures would be required for each time point tested to avoid distorting the results.

Throughout exponential growth of the parental strain 630Δ*erm* the mixed and unmixed cultures had very similar OD_600_s to one another, indicating that during growth of this strain little sedimentation occurs (Fig. 5B). At 24 h however the OD_600_s of the unmixed cultures were lower than the mixed (OD_600_ of approximately 0.8 and 1.2 respectively), suggesting that at stationary phase cells begin to sediment out of solution (Fig. 5B). No difference was observed in the overall growth of the *fliC* mutant compared to 630Δ*erm*. In the unmixed culture of the *fliC* mutant the OD_600_ increases between 3 and 7.5 h, however these OD_600_s are lower than those of both the *fliC* mixed cultures and the 630Δ*erm* unmixed cultures, indicating that some sedimentation occurs in this strain during growth (Fig. 5B). By 24 h the OD_600_ of the unmixed culture fell to an OD_600_ of approximately 0.2, while the mixed culture was around 1.0, this difference in OD_600_ at this time point is much greater than was observed in 630Δ*erm*.

In the modification gene mutants CD0241, CD0242 and CD0244, overall growth was not significantly different to 630Δ*erm* or the *fliC* mutant, however the sedimentation phenotype was much more pronounced and no increase in OD_600_ was observed in the unmixed culture beyond an OD_600_ of approximately 0.2 throughout the exponential growth phase (Fig. 5C). In line with other phenotypic observations, the knock-out mutant of CD0243, which encodes the putative methyltransferase, did not display a phenotype which was significantly different to the parental strain (Fig. 5A and C).

While it appears that the loss of the flagellin results in some sedimentation, mutations in the modification genes cause a more extreme phenotype which is continuous throughout growth. We therefore hypothesized that this phenotype is due to the presence of a flagella filament lacking the full repertoire of modifications. To address this hypothesis we took advantage of the recent progress in genetic tools for *C. difficile* (Faulds-Pain and Wren, 2013) and generated in-frame deletions of the *fliC* gene by allele exchange in the CD0241, CD0242 and CD0244 clostron mutant genetic backgrounds. Destructive growth curves were carried out as before and from the OD_600_ of the unmixed cultures it was found that sedimentation did occur during the growth of all three double mutants (Fig. 5D), however, unlike the single modification gene mutants, which remained at an OD_600_ of approximately 0.2 in the unmixed cultures, the double mutants did increase during growth phase, peaking at OD_600_ of between 0.8 and 1.2 at 7.5 h and had a comparable profile to the *fliC* single mutant (Fig. 5B). This supports the hypothesis that the sedimentation which occurs in these modification mutants is chiefly a consequence of an incompletely modified flagella filament.

**Fig 6:**
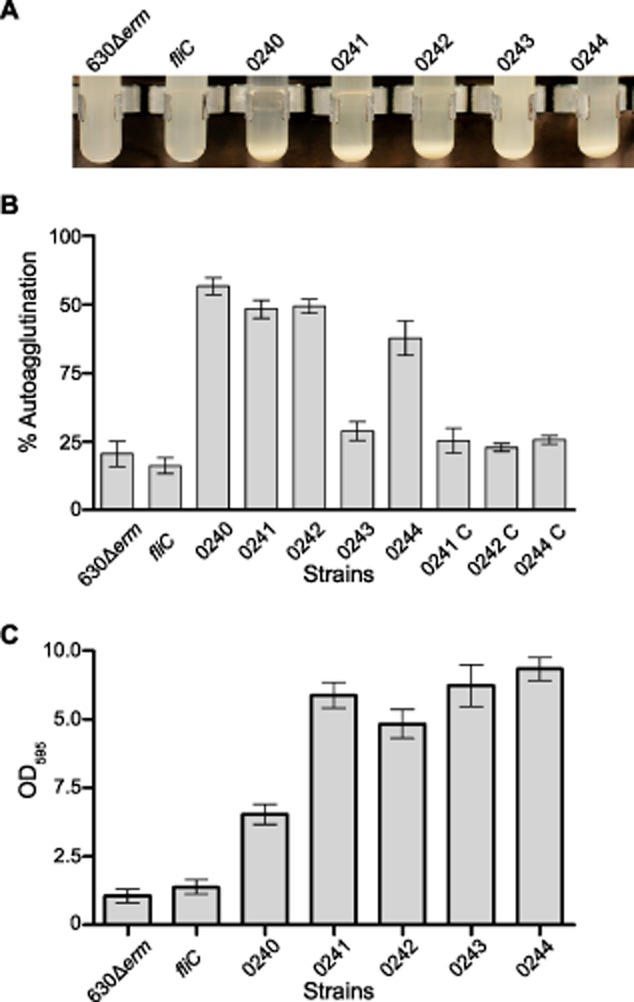
Agglutination of cells to one another and abiotic surfaces.A and B. The autoagglutination of the modification mutants was measured in the absence of media by growing them on solid media and transferring them to PBS. The total autoagglutination was calculated as the OD_600_ of the top 1 ml of cell solution compared to the total OD_600_ following 16 h incubation. Autoagglutination was found to be the highest in CD0240, CD0241, CD0242 and CD0244 mutants and could be restored on complementation, the *fliC* and CD0243 mutant were similar to the parental strain.C. The agglutination phenotype of the mutants was also investigated in their interaction with abiotic surfaces, strains were grown in 24-well plates for 5 days and the relative amount of cells bound was measured with crystal violet stain. All of the modification mutants showed a marked increase in binding to the surface of the wells compared to 630Δ*erm* and the *fliC* mutant.

To determine whether these cells are interacting with one another or with components of the growth medium, the mutants were tested for autoagglutination. Cells were first grown on solid BHIS agar then transferred to PBS, all strains were then normalized by absorbance to a starting OD_600_ of 10, as described previously (Howard *et al*., 2009; Reynolds *et al*., 2011). Following incubation overnight, sedimentation was observed in the CD0240, CD0241, CD0242 and CD0244 mutants (Fig. 6A). The results were quantified by calculating the difference between the absorbance of the top 1 ml of the solution and the absorbance of the whole volume, autoagglutination was calculated as the percentage of cells that had sedimented (Fig. 6B). This was found to be between 10 and 20% in the parental strain, the *fliC mutant* and the CD0243 mutant but 86% in the CD0240 mutant and between 65% and 72% in the CD0241, CD0242 and CD0244 mutants.

### Alterations in the flagella PTMs leads to increased cell binding to abiotic surfaces

The autoagglutination observed in the CD0240, CD0241, CD0242 and CD0244 PTM mutants indicated that these cells had a ‘stickiness’ that caused them to group together and as a consequence settle out of solution. To determine whether this also made them more adherent to abiotic surfaces, *C. difficile* strains were grown in 24-well plates for 5 days and the comparative number of cells adhered to the surface of these plates were quantified by crystal violet staining (Fig. 6C; Dawson *et al*., 2012). All of the PTM mutations lead to an increased interaction with the surface of the plates; interestingly however this phenotype among the modification mutants was the least pronounced in the CD0240 mutant which had the strongest auto-aggregation phenotype. In contrast the CD0243 mutant, for which we previously observed no phenotypes, adhered to the abiotic surfaces as well as the CD0241, CD0242 and CD0244 mutants.

### The role of PTM in the epidemic ribotype 017 strains

**Fig 7:**
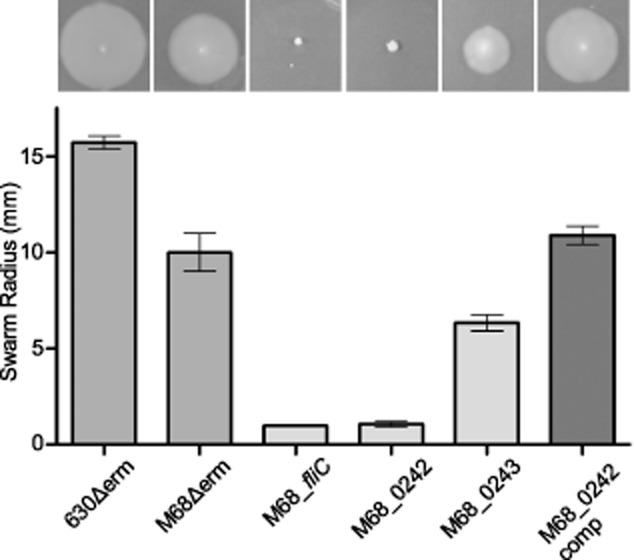
Motility in M68 putative modification mutants. Motility assays of both the 630Δ*erm* and M68Δ*erm* parental strains compared to the M68Δ*fliC* in-frame deletion mutant and the mutations in the predicted modification genes M68_CD0242 and M68_CD0243 were carried out in CDMM containing 0.3% Difco-bacto agar. M68Δ*erm* was motile and the M68_*fliC* mutant non-motile, as described previously. A mutant of the M68_0242 gene was non-motile, this was restored upon complementation. The M68_0243 mutant was motile, with a slightly reduced motility compared to the parent.

The configuration of the 630 type A modification locus was also identified in ribotype 017 strains, a distinct *C. difficile* lineage, members of which have caused a number of epidemics across Europe and Asia, but are toxin A negative (Drudy *et al*., 2007). To determine whether the properties associated with the PTM mutants in 630Δ*erm* could be extrapolated to the orthologues in the ribotype 017 strains, mutants were made in the strain M68Δ*erm*, a erythromycin-sensitive derivative of the ribotype 017 strain M68 isolated from an outbreak in Dublin (Drudy *et al*., 2007; Faulds-Pain and Wren, 2013). An in-frame deletion of the chromosomal *ermB* gene of M68, which we had previously constructed, makes this strain sensitive to macrolide, lincosamide, streptagramin (MLS) antibiotics which are typically required for mutagenesis by Clostron insertion (Faulds-Pain and Wren, 2013). The two genes M68-0242 and M68-0243 (orthologues of CD0242 and CD0243 respectively) were selected for mutagenesis as the former had a strong phenotype in 630Δ*erm* and the latter a subtle phenotype. Motility of these mutants was assayed and compared to 630Δ*erm* and M68Δ*erm* and a *fliC* deletion mutant of M68, described previously (Faulds-Pain and Wren, 2013). As in the 630Δ*erm* background, the M68-0242 mutant caused a complete loss of motility which was restored on complementation, while the M68-0243 mutant was slightly less motile than the parental strain (Fig. 7A and B).

### Flagellin modification is essential for efficient colonization and recurrence in the murine model of infection

In a murine model, *C. difficile* can cause a self-limiting, chronic intestinal infection by which colonization and relapse can be studied (Chen *et al*., 2008; Lawley *et al*., 2009). We used this model to assess the role of the flagellin modifications in the establishment and recurrence of *C. difficile* infection. In order to clear the natural intestinal microflora and allow *C. difficile* to establish an infection, clindamycin was administered to 8 week-old C57BL/6 mice 5 days prior to challenge with *C. difficile* spores derived from either 630Δ*erm* or the isogenic *fliC*, CD0241 or CD0243 mutants. Shedding of *C. difficile* in the faeces was used as an indirect measure of host colonization and was determined by plating emulsified faecal pellets and enumerating the colony forming units (cfu) per gram of faeces. In this model, colonization is transient and detection of bacteria in the faeces is difficult 14 days post infection. However, as low numbers persist, a second dose of clindamycin administered approximately 28 days post infection stimulates outgrowth and shedding, indicating relapse of infection, can be monitored.

**Fig 8:**
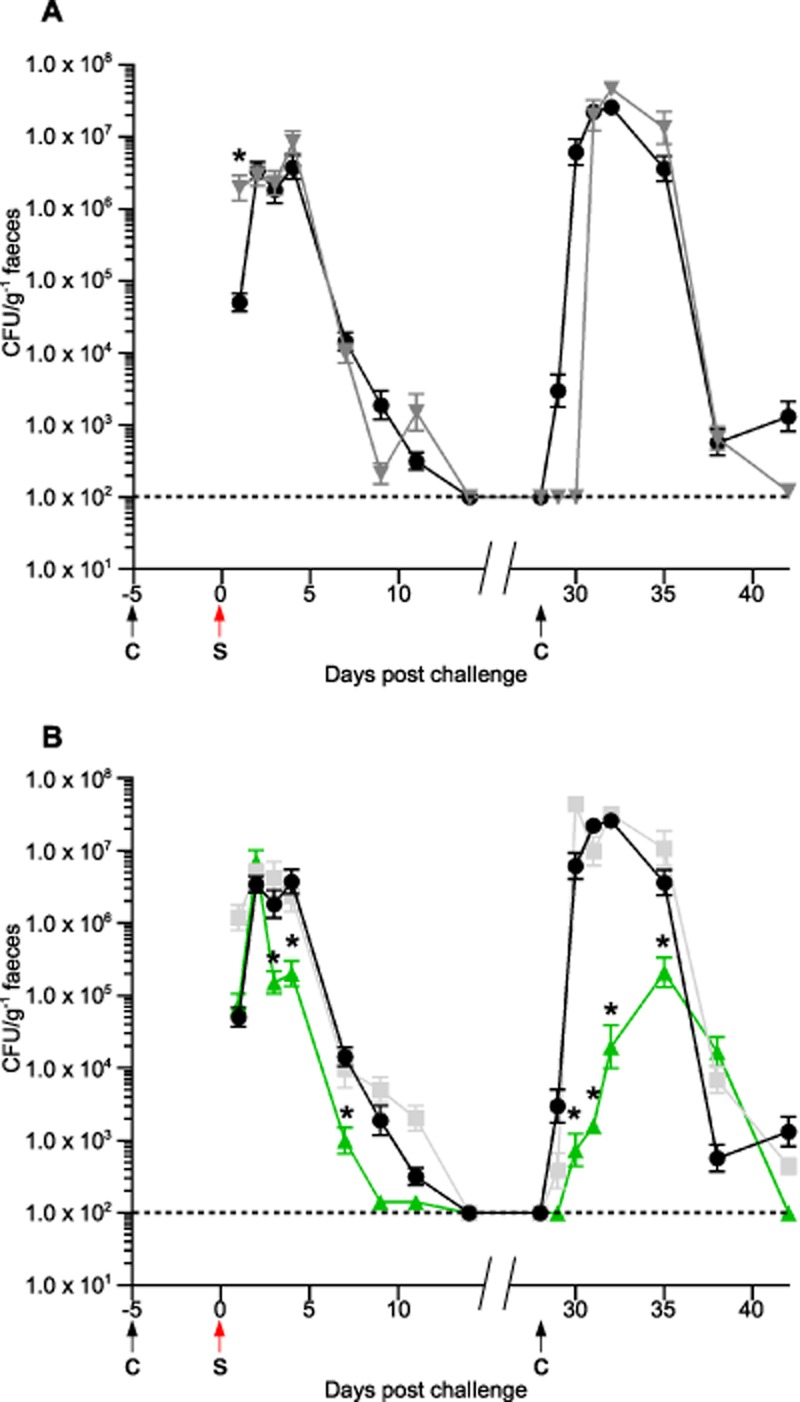
Flagellin modification is essential in the colonization and recurrence of *C. difficile* infection in C57BL/6 mice. Mice were challenged with *C. difficile* spores (S) 5 days following administration of clindamycin (C). Levels of *C. difficile* were measured by plating faecal samples and determining the *C. difficile* cfu g^−1^.A. Mice were challenged with the parental strain 630Δ*erm* (inverted grey triangles) and the non-motile *fliC* Clostron mutant (black circles). Initial colonization is similar in these strains but relapse is delayed in 630Δ*erm* by 2 days following the second dose of clindamycin.B. The initial colonization and relapse of the *fliC* mutant (black circles) were compared to the CD0241 and CD0243 modification mutants (green triangles and light grey squares respectively). While CD0243 has a similar initial colonization and recurrence rate to the *fliC* mutant, the CD0241 mutant is defective in both initial colonization and recurrence. Asterisks indicate a significant difference compared to the *fliC* mutant.

Mice challenged with the parental strain 630Δ*erm* shed the bacteria at high levels from day 1 until day 4 post challenge (approximately 5 × 10^6^ cfu g^−1^ faeces), by day 7 the *C. difficile* load in the faeces had begun to fall (*c.* 1 × 10^4^ cfu g^−1^ faeces) and by day 14 the load of *C. difficile* in the faeces was below the limit of detection (Fig. 8A). Recurrence of *C. difficile* shedding occurred 3 days after the administration of a second dose of clindamycin (experiment day 31). Shedding peaked after 4 days (day 32) (approximately 4.6 × 10^7^ cfu g^−1^ faeces) and remained high at 7 days (day 35), after 14 days (day 42) levels had dropped almost to the level of detection of the experiment.

In these experiments mice challenged with the *fliC* mutant both the initial infection and the relapse of infection had a similar profile to the parental strain (Fig. 8A). The most significant difference between these two strains was that following the second dose of clindamycin *C. difficile* shedding in the faeces recurred after 1 day in the mutant (approximately 3 × 10^3^ cfu g^−1^ faeces) compared to 3 days in the parental strain. After 4 days (experiment day 32) shedding of the *fliC* mutant peaked (approximately 2.6 × 10^7^ cfu g^−1^ faeces) and remained high at 7 days (day 35), as was observed in the parental strain. At 14 days (day 42) *C. difficile* was still detectable in the faeces but in low numbers (approximately 1.2 × 10^3^ cfu g^−1^ faeces). The shedding profile of mice challenged with the CD0243 mutant was not significantly different to strain the *fliC* mutant at any point post challenge or post second clindamycin treatment (Fig. 8B). The delay in re-colonization following the second dose of clindamycin observed in the parental strain did not occur in any of the mutants tested. As the Clostron mutants are marked with an *ermB* gene, which confers resistance to clindamycin [minimum inhibitory concentration (MIC) *fliC* = 16 μg ml^−1^, CD0241 = 8 μg ml^−1^ and CD0243 = 4 μg ml^−1^], while the parental strain, 630Δ*erm* is sensitive to this class of antibiotics (MIC = 0.25 μg ml^−1^), it is likely that the clindamycin in the mice inhibits the recurrence of 630Δ*erm* but not the mutants until the residual antibiotic is cleared.

Mice infected with spores of the CD0241 mutant reached a similar level of shedding as the parental strain by day 2 post challenge; however, while the parental strain and the *fliC* and CD0243 mutants continued to shed at high levels until day 4, shedding of the CD0241 mutant fell on day 3 and remained significantly lower than the *fliC* mutant at day 4 (*P* = 0.04; Fig. 8B). This trend continued at day 7 and at both days 9 and 11 the number of *C. difficile* cfu in the faeces was almost at the limit of detection. Following the second dose of clindamycin, recurrence of infection by the CD0241 mutant did occur, however the shedding of this mutant in the faeces was significantly less than the *fliC* mutant at all time points tested up to 10 days later (day 38) (*P* = 0.0011).

Weight change was monitored in the mice as another marker of infection. The animals were weighed before challenge and at 2 days post challenge with all four strains. Those infected with 630Δ*erm*, the *fliC* mutant and the CD0243 mutant all lost weight in this time (between 2.3% and 6.3% of total body weight) compared to those challenged with the CD0241 mutant (gained 5% body weight). The difference in the change of weight in animals challenged with this strain was significantly different than 630Δ*erm* (*P* = 0.002), the *fliC* mutant (*P* = 0.008) and the CD0243 (*P* = 0.01) mutant. It was also observed that the mice challenged with either 630Δ*erm*, the *fliC* mutant and the CD0243 mutant produced sticky faeces in their cages, a sign of significant infection, this was not observed in those animals challenged with the CD0241 mutant.

In this experiment colonization of C57BL/6 mice and disease recurrence does not appear to be reliant on bacterial motility or the presence of the flagellin in *C. difficile* strain 630Δ*erm*; however, disruption of the flagellin modification leads to attenuation in both colonization and relapse.

## Discussion

Treatment of *C. difficile* disease is extremely difficult and relapse rates are high. Much work has been dedicated to the study of *C. difficile* toxins as they are important in disease and to sporulation and the spread of infection. However, little is known about the organisms' ability to interact with and colonize the intestinal epithelia of the host, a trait which may be vital in disease recurrence. In this study, we defined the structure of the type A flagellin post-translational modification by NMR and found that the resultant change altered the extent of colonization and outgrowth in the murine relapse model of infection.

Of the known flagellin modification structures only some *P. aeruginosa* strains produce a structure composed of a sugar plus a phosphate and a methylated amino acid, similar to the *C. difficile* type A flagellin modification (Verma *et al*., 2006). Interestingly, as with *C. difficile*, the *P. aeruginosa* flagellins are also subject to strain-specific variation of the PTMs, some being modified as the *C. difficile* type A strain such as strain PA01 and others, such as the strain PAK being modified with a larger glycan composed of a number of monosaccharide moieties (Verma *et al*., 2006). The precise role of this modification in *P. aeruginosa* has not been determined; however, flagella are known to be essential in the establishment of acute infections by *P. aeruginosa* (Cobb *et al*., 2004). The *C. difficile* type A flagellin modification was previously studied by LC-MS/MS and the initial structure was assigned as a HexNAc sugar with a methylated aspartic acid attached via a phosphate bond (Twine *et al*., 2009). On further analysis however it was realized that this amino acid was mis-identified as an aspartic acid, the mass of the moiety attached to the phosphate group being 115 Da is too small for an aspartic acid plus a methyl group. In this study, to definitively determine the structure of this modification a large-scale extraction of *C. difficile* 630 flagellin was completed. The flagellin protein was extensively digested with proteinase K and following purification the glycan structure was solved by NMR. The structural configuration of the HexNAc linking sugar was confirmed to be β-O linked GlcNAc and the remaining modification was linked via phosphate bond and determined to be an *N*-methylthreonine residue in the l-configuration.

Oligosaccharide flagellin modifications are commonly formed of a series of sugars, predominantly pseudaminic acid or its derivative legionaminic acid (Asakura *et al*., 2010; Parker *et al*., 2012; Sun *et al*., 2013). Specialist enzymes are required for the biosynthesis of these sugars as they are not synthesized by the cell for any other purpose (Parker *et al*., 2012; Sun *et al*., 2013). This is not the case for the *C. difficile* type A flagellin modification, as GlcNAc sugars are common precursors for many biosynthetic pathways, therefore no specialized biosynthesis genes are required. This is reflected in its modification genes (Fig. 1), which are predicted to be involved in the transfer of the modifications rather than the synthesis of precursors.

**Fig 9:**
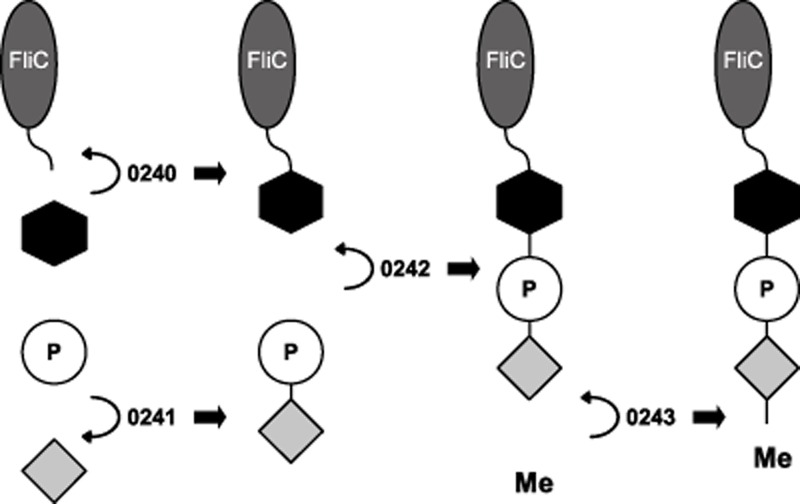
Schematic diagram illustrates the structure of the type A modification and the predicted contribution of the enzymes to the synthesis of the glycan. The FliC protein is modified at seven serine and threonine residues (grey oval represents a FliC monomer with the curved line representing either a serine or threonine amino acid within the FliC protein). To these residues a GlcNAc (black hexagon) is transferred by the GT encoded by CD0240. A phosphate group (white circle) is attached to a threonine (light grey diamond) by the enzyme encoded by CD0241 and this phosphorylated threonine is attached to the FliC-GlcNac by the enzyme encoded by CD0242. The methyltransferase encoded by CD0243 adds a methyl group to the threonine.

By generating mutations of the *C. difficile* type A modification genes and studying the resulting structures on the flagella filaments by LC-MS/MS, we were able to define more specifically the role each gene plays in the resulting modification. Flagellins of the CD0241 and CD0242 mutants are modified with the GlcNAc sugar only and both lack the phosphate group and the *N*-methylthreonine residue (Fig. 2). This indicates that the addition of the phosphate and the threonine to the GlcNAc is not sequential, but rather that they are added to the structure together. These genes encode proteins which have similarity with phosphoserine phosphatases and phosphocholine cytidylyltransferases respectively. Phosphoserine phosphatases catalyse the reversible reaction of serine and phosphate to phosphoserine and H_2_O, and it seems likely that CD0241 could encode a kinase which catalyses the addition of phosphate to threonine (Fig. 9). This is supported by a DXDX(T/V) motif encoded at the N-terminus of CD0241, this domain functions as an intermediate phosphoryl acceptor, indicative of a phosphatase (Osman *et al*., 2010). Phosphocholine cytidylyltransferases catalyse the reversible reactions of CTP plus choline phosphate to CDP-choline and diphosphate. As there is no evidence of phosphocholine in the flagellin modification, we hypothesize that the enzyme encoded by CD0242 catalyses the addition of the phosphothreonine to the GlcNAc residue (Fig. 9).

LC-MS/MS of the putative methyltransferase knockout (CD0243) identified a mixed population of glycan structures on flagellar peptides; either the GlcNAc residue alone or the extended structure where the threonine was no longer methylated. The former population indicates that the function of this enzyme is to transfer a methyl group to the threonine. This is represented as the final step in the formation of the glycan in Fig. 9, however from our data we cannot discern at what stage methylation occurs, whether it is when the threonine is in complex with the glycan, when it phosphorylated or when it is free threonine. The presence of some residues which are modified with GlcNAc alone is less easy to interpret; but it may indicate that when the methyl group is absent the modification is less stable. It is also unclear from our data what the function of the fourth gene in the locus, CD0244, might be. The LC-MS/MS analysis identifies a mixed population of both the wild-type structure and the GlcNAc alone; however the phenotypic characteristics of this mutant are the same as the CD0241 and CD0242 mutants. This predicted protein has some similarity with glycerophosphotransferase enzymes; consequently, we would predict that it is involved in the transfer of the phosphate group to either the threonine or the GlcNAc. This mixed population may indicate that this enzyme is partially redundant. Interestingly, the similar flagellin PTM cluster in *P. aeruginosa* PA01 strains contains only orthologues of CD0241, CD0242 and CD0243, with CD0244 absent, supporting this redundancy hypothesis. This protein may however have a function not identified in this current study.

Previously, the CD0240 mutant, predicted to encode the initial glycosyltransferase enzyme, was characterized and found to be non-motile (Twine *et al*., 2009). Unusually for strains with a mutation that prevents flagellin modification, this knock-out was still able to both secrete flagellin and form a filament. In this study we found that the CD0241, CD0242 and CD0244 mutants likewise produce flagella and are non-motile, but the mutant of CD0243 retained motility similar to the parental strain. Loss of motility in flagellin PTM mutants in most organisms can be explained by the absence of a flagella filament, this is not the case for the *C. difficile* PTM mutants suggesting that other properties of these unmodified or partially modified filaments are interfering with motility. We noted another property of these mutants was their tendency to aggregate to each other and to surfaces and consequently to sediment when grown in broth. This phenotype was independent of motility and was dependent on the presence of an unmodified or incompletely modified flagella filament. One possible explanation is that an alteration in the flagellin modifications affects the polarity of the cell surface, causing the aggregation of the cells which prevents them from swimming efficiently. An alternative hypothesis is that the alteration in the charge on the flagellins is causing deleterious changes in the regularity of filament pitch height and length and consequently affecting the organisms' ability to swim efficiently.

The type A modification gene locus identified in 630 has also been identified in the RT017 epidemic lineage of *C. difficile*. We found that the phenotypes associated with the type A modification mutants in 630Δ*erm* were also associated with mutants of their orthologues in the RT017 strain M68Δ*erm*.

Little is known about the advantages flagellin PTMs provide a bacterium, although they have been implicated in host colonization and virulence in a number of organisms (Balonova *et al*., 2012; Iwashkiw *et al*., 2012; Lithgow *et al*., 2014). Flagellin is a hugely abundant protein, prominently displayed on the surface of the cell and PTMs are localized to its externally exposed domains, indicating that their function may lie in the interaction with the environment. C57BL/6 mice have been shown to be a useful model for the study of *C. difficile* colonization and recurrence as they do not succumb to the overwhelming effects of the toxins during infection (Chen *et al*., 2008; Lawley *et al*., 2009). To investigate the role of flagellin, motility and PTMs in the infection and relapse, these mice were challenged with the parent strain, 630Δ*erm*, and the *fliC*, CD0241 and CD0243 mutants. We found that the non-motile *fliC* mutant and the motile CD0243 mutant had the same profile of faecal *C. difficile* loads as 630Δ*erm* during both the initial infection and relapse, although the relapse of clindamycin sensitive 630Δ*erm* was delayed by 2 days compared to the mutants. The Clostron mutagenesis method used to generate our mutants requires an MLS antibiotic sensitive parent strain, in this case 630Δ*erm*, and results in mutants marked with an *ermB* gene which confers MLS antibiotic resistance. As clindamycin is a MLS antibiotic and in this experiment it is used to trigger relapse, it is likely that the delay in 630Δ*erm* recurrence is due to its sensitivity to clindamycin and the ability of the mutants to immediately re-grow is due to their resistance. During the initial infection the non-motile CD0241 mutant reached the same peak of bacterial load in the faeces as the other three strains however, this decreased much sooner and relapse of infection was slower, peaked later and reached a lower bacterial load in the faeces. These mice also avoided other symptoms of severe infection such as weight loss and sticky faeces.

We observed that neither flagella nor motility appear to be required for the efficient colonization of *C. difficile* in the mouse intestine, as had been described previously (Baban *et al*., 2013). The complete modification of the flagellin however does appear to be important in colonization and relapse. It is not known whether the CD0241 mutant aggregates *in vivo* as it does *in vitro* but it is possible that this phenotype is affecting the fitness of the bacteria or the hosts' ability to clear it from the intestine. Potentially the alteration in the modification structure could affect the interaction of this highly abundant protein with the hosts' immune system.

## Experimental procedures

### Bacterial strains and growth conditions

*Escherichia coli* Top10 (Invitrogen) and *E. coli* CA434 were grown at 37°C in Luria–Bertani (LB) growth media supplemented with chloramphenicol (12.5 μg ml^−1^) and CA434 with Kanamycin (50 μg ml^−1^) where appropriate. *C. difficile* was cultured at 37°C in an anaerobic work station (Don Whitley, Yorkshire, UK) and grown routinely in BHIS medium (Brain heart infusion medium supplemented with 5 mg ml^−1^ yeast extract and 0.1% w/v l-cysteine). *C. difficile* was activated from glycerol stocks on Braziers agar [CCEY (Bioconnections), 4% w/v egg yolk and 1% w/v defibrinated horse blood]. *C. difficile* was supplemented with d-cycloserine (250 μg ml^−1^), cefoxitin (8 μg ml^−1^), thiamphenicol (15 μg ml^−1^) and erythromycin (10 μg ml^−1^) where appropriate. Plasmids were transferred to *C. difficile* by conjugation from *E. coli* CA434 as described previously (Heap *et al*., 2007; Cartman and Minton, 2010).

*C. difficile* minimal media (CDMM) (Cartman and Minton, 2010) containing 0.3% bacto-agar (DIFCO) was used to assay motility of *C. difficile* strains, plates containing 35 ml media were inoculated with a single colony using a sterile toothpick and incubated for 3 days. Images were captured using a Canon 600D SLR camera. Minimum inhibitory concentration (MIC) assays were performed as described previously (Andrews, 2001).

### General molecular biology techniques

Plasmids were extracted using a plasmid mini kit (Qiagen) and the genomic DNA with the DNeasy Blood and tissue kit (Qiagen) following treatment with lysozyme (10 mg ml^−1^ in phosphate-buffered saline at 37°C for 30 min), then SDS (10% w/v) at 65°C for 30 min (Cartman and Minton, 2010) or Chelex extraction [cell pellets vortexed in 5% chelex (Sigma) boiled for 10 min, pelleted and the supernatants removed and used] or by phenol-chloroform purification as described previously (Cartman and Minton, 2010). DNA was amplified for cloning using Phusion high fidelity polymerase (NEB), and for screening using Go-taq polymerase (Promega), both in accordance with the manufacturers' instructions. DNA was extracted from PCR reactions and agarose gels using the QIAquick PCR and gel extraction kits (Qiagen) respectively. Allele exchange and complementation plasmids were constructed by restriction/ligation cloning using restriction endonucleases, Antarctic phosphatase and T4 ligase (NEB). Clostron plasmids were designed at http://www.clostron.com and synthesized and cloned into pMTL007C-E2 by DNA2.0 (Heap *et al*., 2010).

Southern blot analysis was done using an intron specific probe. One microgram of genomic DNA was digested overnight with HindIII restriction enzyme (Promega). AlkPhosDirect™ Labelling and detection kit (GE Healthcare) and detection reagents, in accordance with the manufacturer's guidelines and visualized using CDP star (GE Healthcare).

### Mutagenesis

*C. difficile* was mutated using the Clostron mutagenesis system as described previously (Heap *et al*., 2007; 2010[Bibr b20],[Bibr b22]). Briefly, gene-specific re-targeted Clostron plasmids (Table 2) were transformed in to *C. difficile* by conjugation and transconjugants selected for with thiamphenicol, mutants were then positively selected with erythromycin and confirmed by PCR and Southern blot. Double mutants were made in *C. difficile* by allele exchange using the method described previously (Faulds-Pain and Wren, 2013). Briefly, the plasmid pAFP91 (Faulds-Pain and Wren, 2013) was transformed in to the *C. difficile* 630Δ*erm* Clostron mutants of CD0241, CD0242 and CD0244 by conjugation and transconjugants selected for with thiamphenicol, a series of replica plating on thiamphenicol allowed single crossover integrants to be isolated, double crossovers were then isolated by replica plating without selection and identified by their inability to grow on thiamphenicol. Mutant construction was verified by PCR.

### Construction of complementation vectors

The *fliC* complementation vector contained the genes own promoter and terminator, the primers amplified from the end of the TAA stop sequence of the gene immediately upstream of the *fliC* and 196 bp downstream of the *fliC* stop site. The PCR product was cloned into the *C. difficile* complementation vector pMTL84151 (Heap *et al*., 2009; Table S1 for primers and restriction sites). The CD0241 and CD0242 ORFs only were amplified and cloned into the complementation vector pMTL84153 (as the pMTL84151 vector but with an *fdx* promoter/RBS and terminator) at the NdeI site, immediately after the RBS in the vector and utilizes the ATG in its restriction sequence. The CD0244 ORF already contains an NdeI restriction site within its sequence therefore to utilize this restriction site as with the other two ORFs, the internal NdeI site was first removed by making a non-coding change in the sequence by SOE-PCR. All constructs were confirmed by restriction analysis and Sanger sequencing (SourceBioscience). In these three vectors expression of the complement genes are driven by the non-native constitutive promoter from the *fdx* gene of *Clostridium pasteurianum*.

### Sedimentation growth curves

Sixty millilitres of BHIS broth was inoculated with a 1:100 dilution of an overnight culture, the inoculated broth was split into 5 ml aliquots which were each allowed to grow statically. At specific time points the top 1 ml of a 5 ml culture was carefully removed from one replicate, while the second replicate was mixed by vortexing and 1 ml of this mixed culture was removed, the OD_600_ of both were measured. These cultures were then disposed of and the subsequent OD_600_ reading taken from another pair of 5 ml cultures. Assays were repeated in triplicate. Statistical analysis was done in GraphPad Prism by two-way anova and groups compared using a Tukey's multiple-comparisons test.

### Autoagglutination assay

Strains of *C. difficile* grown overnight on BHIS agar were resuspended in PBS and normalized to an OD_600_ of 10 in a volume of 5 ml. Following incubation for 16 h at 37°C in the anaerobic cabinet the top 1 ml of cells was removed and the OD_600_ measured and the OD_600_ of the whole solution measured. Autoagglutination was calculated as the difference between the OD_600_ of the whole solution and the OD_600_ of the top 1 ml as a percentage of the whole solution OD. Assays were repeated in triplicate.

### Adherence to an abiotic surface

Overnight cultures of *C. difficile* were used to inoculate 2 ml of BHIS in high bind 24-well plates at a 1:100 dilution. The cultures were grown for 6 days after which the supernatant was carefully removed and the wells washed with PBS. 1% crystal violet was added to the wells and incubated at room temperature for 20 min, the wells were then washed twice with PBS and the remaining crystal violet was detached from the cells attached to the surface of the wells with methanol. The OD_595_ was measured in a Spectrophotometer (Biotek, UK). Assays were repeated in triplicate.

### Transmission electron microscopy

Cultures of *C. difficile* were inoculated 1:100 from overnight culture and allowed to grow statically for the 6 h. An equal volume of fixative (2.5% paraformaldehide, 2.5% glutaraldehide, 0.1 M Na cacodylate at pH 7.4) was added to the cultures. Fixed samples were diluted in MilliQ water at a 1:6 dilution and 10 μl spotted on to a 400 mesh copper grid with a pioloform support film and incubated for 1 min. Excess liquid was then removed by pipetting and 10 μl of 0.3% phosphotungstic acid (PTA) added in order to stain the sample and incubated for 1 min. The excess PTA was drawn off with filter paper and the grid air-dried before examining on a Jeol JEM-1200EX II Transmission Electron Microscope fitted with a 2K side mounted AMT (Advanced Microscopy Techniques) CCD digital camera supplied by Debens UK Ltd (http://www.debens.co.uk), used to record the digital images.

### Low-pH glycine extractions

Cells from 18 h overnight cultures of *C. difficile* strains were harvested, washed with phosphate buffered saline and resuspended in a 1:100 volume of low-pH glycine (0.2 M glycine-HCl, pH 2.2) and incubated at room temperature for 20 min with gentle shaking. The cells were removed by centrifugation at 4°C and the supernatant neutralized with the addition of 2M Tris to a pH of 7 to 8.

### Purification of flagellar glycan for NMR analysis

Flagellar glycoprotein sample was digested with a large excess of proteinase K in 0.01 M TRIS-HCl buffer at pH 8 at 37°C for 48 h. The products of digestion or free oligosaccharides were separated on Bio-Gel P6 column (2.5 × 60 cm) and each fraction which eluted before the salt peak was dried and analysed by ^1^H NMR. Fractions containing sugars were separated by anion exchange chromatography on Hitrap Q column (5 ml size, Amersham) and the glycans eluted with a linear gradient of NaCl (0–1 M, 1 h). Desalting was performed on Sephadex G15 prior to analysis by NMR.

### NMR spectroscopy analysis

NMR experiments were carried out on a Varian INOVA 500 MHz (^1^H) spectrometer with 3 mm gradient probe at 25°C with acetone internal reference (2.225 ppm for ^1^H and 31.45 ppm for ^13^C) using standard pulse sequences DQCOSY, TOCSY (mixing time 120 ms), ROESY (mixing time 500 ms), HSQC and HMBC (100 ms long range transfer delay). AQ time was kept at 0.8–1 s for H-H correlations and 0.25 s for HSQC, 256 increments was acquired for t1. For ^1^H-^31^P HMQC H-P coupling was set to 10 Hz.

### Absolute configuration of *N*-methylthreonine

Glycopeptides were dephosphorylated with 48% HF (10 μl ml^−1^, 30 min, 30°C), dried by air stream, (*R*)-2-octanol (0.2 ml) and acetyl chloride (0.02 ml) were added at room temperature, heated at 100°C for 2 h, dried by air stream, acetylated (0.2 ml Ac_2_O–0.2 ml pyridine, 100°C, 30 min), dried, and analysed by GC-MS as described above. A standard was prepared from *N*-methyl-l-Thr (Sigma) by the same procedure without the dephosphorylation step with (*R*)- or (*RS*)-2-octanol. Analysis was performed on Agilent 6890 GC instrument with DB17 column at 160–280°C by 4°C min^−1^.

### In-gel digestion of flagellin proteins for mass spectrum analysis

Flagellin gel bands were excised and destained using 25% ethanol: 10% acetic acid. The gel bands were subsequently dehydrated with the addition of acetonitrile. Fully dehydrated gel bands were air dried under a laminar flow hood and then digested over night with 20–50 μl of 20 ng μl^−1^ sequencing grade modified trypsin in 50 mM Ammonium bicarbonate. Digests were incubated at 37°C overnight. Peptides were extracted into fresh microfuge tubes and stored at −20°C.

### Mass spectral identification and *de novo* sequencing of in-gel digested flagellins

Protein digests were analysed using a QTOF Ultima coupled to a nanoAcquity UPLC system. Briefly, 10 μl of each digested was injected onto a 180 μm ID × 20 mm, 5 μm symmetry C18 trap column in trapping mode, diverting flow through to waste. Peptides were eluted by reversed phase liquid chromatography (RPLC) to a 100 μm ID × 10 cm, 1.7 μm BEH130 C18 column (Waters) in analytical mode, using a linear gradient from 1% to 45% solvent B in 18 min, 45% to 85% solvent B for 2 min, 85% to 1% solvent B over 1 min, and hold for 8 min at 1% solvent B. Tandem mass spectrometry analyses were performed in data-dependent acquisition (DDA) mode. Peaklist files were generated and searched against the *C. difficile* 630 translated genome sequence, using MASCOT (Matrix Science) to identify unmodified peptides. The mass tolerance for precursor and fragment ions was 0.8 Da and an ion score of 30 or above indicated identity. In-house software ‘Glycan Hunter’ performed peak listing of MS/MS spectra of unmatched spectra that featured key glycan related ions, using information from previously published studies (Twine *et al*., 2009). Glycopeptides were identified by manual inspection of the MS/MS data, and *de novo* sequencing of peptide related ions.

### Western blot

The flagellin was isolated by low-pH glycine extractions in all cases. For the anti-FliC Western blots the low-pH glycine extraction was carried out as described above. Samples were normalized according to the OD_600_ of the cultures and a total volume of 15 μl was run on a Nu-Page 12% Bis-Tris SDS-PAGE gel in MOPS running buffer (both Life Technologies). Proteins were transferred to an H + membrane in a semi-dry blotter in transfer buffer (0.24% Tris, 1.14% glycine and 20% Methanol). The membrane was blocked in PBS plus 2% milk (PBS-M) and probed with an anti-FliC antibody (a generous gift from the Armstrong lab) at a 1 in 10 000 dilution for 1 h at room temperature. Following standard membrane washing with PBS-T (PBS plus 0.1% Tween 20) a Li-cor Donkey anti-chicken IR Dye 680RD secondary antibody (Li-cor) was added at a 1: 5000 in PBS-M plus 0.1% Tween 20 and 0.01% SDS and incubated in the dark for 1 h. The membrane was washed as before and visualized using a Li-cor Odyssey.

For the anti-GlcNAc Western blots, low-pH glycine extraction was achieved by scraping overnight growth of *C. difficile* from plates and incubating the cells with low-pH glycine buffer. Samples were standardized according to the OD_600_ of the resuspended cells and protein separated on 12% Tris Glycine gels, and transferred to a PVDF membrane. The membrane was blocked with 5% milk-PBS-T then probed with 1 in 5000 dilution of anti-β-*O*-GlcNAc (Covance, Montreal, Canada) in PBS-T for 45 min at room temperature. After washing with PBS-T, reactivity was detected with anti-mouse IgM HRP conjugate (Sigma Aldrich, Oakville, Canada) secondary antibody at 1 in 10 000 dilution in PBS-T for 45 min at room temperature. Blots were imaged with ECL Prime Western blotting detection kit (GE Healthcare, Baie D'Urfe, Canada) according to manufacturer's guidelines, followed by exposure to X-ray film.

### Ethics statement

All procedures were strictly conducted according to the requirements of the Animals (Scientific Procedures) Act 1986 approved by the Home Office, UK (project licence 60/4218).

### Animal experiments

A total of 55 8-week-old C57BL/6 mice were housed in groups of 5/6 a week before dosing with 150 mg kg^−1^ clindamycin by oral gavage. Five days later animals were challenged with ∼ 1 × 10^5^ spores/mouse *C. difficile* by oral gavage and mouse weight and body condition monitored throughout the study. Cages were changed weekly and the cabinet washed with perasafe after each group. To monitor *C. difficile* shedding faecal pellets from individuals were collected, serially diluted and plated on CCEY agar supplemented with cycloserine/cefoxitin, egg yolk emulsion and 15 μg ml^−1^ lincomycin (for mutants only). At 28 days post challenge, once *C. difficile* shedding levels were below the limit of detection, mice were given a second dose of clindamycin (150 mg kg^−1^) and shedding monitored for infection relapse. Results are expressed as mean ± SE of at least 10 animals from two independent biological replicates. All statistical analyses were performed using the GraphPad Instat 3.10 (GraphPad Instat Software). A Mann–Whitney analysis of variance analysis (anova) was used to determine significant difference in bacterial recoveries between all time points examined. *P*-values ≤ 0.05 were considered significant.

PCR was performed on colonies obtained from faecal shedding to determine the validity of the shedding strain. For the clostron mutants, gene-specific primers in combination with the universal EBS primer was used in PCR reactions, while for 630Δ*erm* MLVA PCR analysis was used to confirm the identity of the shedding of this strain (Table S1). PCRs were done 1 day post challenge and weekly thereafter.
