# Supplementary material for: The post-translational modification of the Clostridium difficile flagellin affects motility, cell surface properties and virulence
Source: Mol Microbiol. 2014 Sep 15;94(2):272–89. doi: 10.1111/mmi.12755 (PMC4441256; doi:10.1111/mmi.12755)
Supplement: Supplementary file 1 — Supporting information [file mmi0094-0272-sd1.pdf]

Supplementary Table. List of primers

Primer name	Sequence (5’-3’)	Function (restriction site)
630 CD0241CT screen F	CAAGAAATACTTCCTGAGATTTCTAAGTG	PCR screen CD0241 mutant
630 CD0241CT screen R	CCAATTCCAATATCAGCATTCTCTATCAT	PCR screen CD0241 mutant
630 CD0242CT screen F	CAATTGTTGTAAACTGTGCAGGTAGAGGA	PCR screen CD0242 mutants
630 CD0242CT screen R	ATCACCATCTAGTGATATAACAGTGTCTC	PCR screen CD0242 mutants
630 CD0243CT screen F	ATATTCCAGAGAATCCCTATATTTCAGT	PCR screen CD0243 mutants
630 CD0243CT screen R	TTTCTTCATATTCTTCTATAGTTCTATATATAACA	PCR screen CD0243 mutants
630 CD0244CT screen F	GATGAGCAGTGCGACTGTTATGTAGTT	PCR screen CD0244 mutant
630 CD0244CT screen R	TCAATTTCATCAATGACACCATCTTTG	PCR screen CD0244 mutant
630 Δ*fliC* screen F	AGTAGTCAATGTAGTCGAAGAT	PCR screen Δ*fliC* mutants
630 Δ*fliC* screen R	GAATTTGGAATCTCTCTATAATAG	PCR screen Δ*fliC* mutants
M13F	ACTGGCCGTCGTTTTACA	PCR screen
M13R	CAGGAAACAGCTATGACC	PCR screen
630 fliC comp F	ACTTAGCCTGAATTCAGTTATAGATTAACTTGTCCG	Amplify 630 *fliC* plus promoter (EcoRI)
630 fliC comp R	AGTACGGATGGATCCTAAGTTCACTCTCTATTGA	Amplify 630 *fliC* plus promoter (BamHI)
630 CD0241 comp F	TGTGTAATTTTTAAGGAGGTGTGTTACATATGGAAAATTATAA ATTTGTTTTTGA	Amplify CD0241 (NdeI)
630 CD0241 comp R	TAGAGGATCCCCGGGTACCGAGCTCGAATTCTTACAACAATTG TTTTAGTAGTTGGCA	Amplify CD0241 (EcoRI)
630 CD0242 comp F	TGTGTAATTTTTAAGGAGGTGTGTTACATATGCCAACTACTAAA ACAATT	Amplify CD0242 (NdeI)
630 CD0242 comp R	GAGGATCCCCGGGTACCGAGCTCGAATTCTTATTTTTTGTTCCT ATACCATTT	Amplify CD0242 (EcoRI)
630 CD0244 comp F1	GTGTGTTACATATGAATCTGATGAACTATAATAA	Amplify CD0244 (NdeI)

630 CD0244 comp R1	GAAAATCATTACCTTCATAAGTATATATCTGAGT	Amplify CD0244 (SOE primer to remove NdeI)
630 CD0244 comp F2	ACTCAGATATATACTTATGAAGGTAATGATTTTC	Amplify CD0244 (SOE primer to remove NdeI)
630 CD0244 comp R2	ATAATATAAGAATTCCTAAATATGGTTATTTAAAAC	Amplify CD0244 (EcoRI)
MLVA primer set	(Marsh *et al.*, 2006)	Screening for 630Δ*erm* in mice
GRD/P/103F	GTACCTGAAACATTAGTTCCATAACTTGCTCC	630Δ*erm-fliC* screening in mice
GRD/P/104F	GTATATTTATACTTAGGAAAGGCTAAGAAGTGTG	630Δ*erm*-0240screening in mice
GRD/P/105F	CAAGAAATACTTCCTGAGATTTCTAAGTG	630Δ*erm*-0241::CT screening in mice
GRD/P/107F	ATATTCCAGAGAATCCCTATATTTCAGT	630Δ*erm*-0243::CT screening in mice
EBS	CGAAATTAGAAACTTGCGTTCAGTAAAC	Screening in mice

CT = clostron; bp = base pairs; F = Forward; R = Reverse. Underlined sequences correspond to recognition sequences for restriction endonucleases
